# Crowdsourced privacy-preserved feature tagging of short home videos for machine learning ASD detection

**DOI:** 10.1038/s41598-021-87059-4

**Published:** 2021-04-07

**Authors:** Peter Washington, Qandeel Tariq, Emilie Leblanc, Brianna Chrisman, Kaitlyn Dunlap, Aaron Kline, Haik Kalantarian, Yordan Penev, Kelley Paskov, Catalin Voss, Nathaniel Stockham, Maya Varma, Arman Husic, Jack Kent, Nick Haber, Terry Winograd, Dennis P. Wall

**Affiliations:** 1 Department of Bioengineering, Stanford University, Stanford, CA USA; 2 Research Scientist, Amazon, Seattle, WA USA; 3 Department of Pediatrics (Systems Medicine), Stanford University, Stanford, CA USA; 4 Department of Biomedical Data Science, Stanford University, Stanford, CA USA; 5 Department of Computer Science, Stanford University, Stanford, CA USA; 6 Department of Neuroscience, Stanford University, Stanford, CA USA; 7 Graduate School of Education, Stanford University, Stanford, CA USA; 8 Department of Psychiatry and Behavioral Sciences (By Courtesy), Stanford University, Stanford, CA USA

**Keywords:** Translational research, Information technology

## Abstract

Standard medical diagnosis of mental health conditions requires licensed experts who are increasingly outnumbered by those at risk, limiting reach. We test the hypothesis that a trustworthy crowd of non-experts can efficiently annotate behavioral features needed for accurate machine learning detection of the common childhood developmental disorder Autism Spectrum Disorder (ASD) for children under 8 years old. We implement a novel process for identifying and certifying a trustworthy distributed workforce for video feature extraction, selecting a workforce of 102 workers from a pool of 1,107. Two previously validated ASD logistic regression classifiers, evaluated against parent-reported diagnoses, were used to assess the accuracy of the trusted crowd’s ratings of unstructured home videos. A representative balanced sample (N = 50 videos) of videos were evaluated with and without face box and pitch shift privacy alterations, with AUROC and AUPRC scores > 0.98. With both privacy-preserving modifications, sensitivity is preserved (96.0%) while maintaining specificity (80.0%) and accuracy (88.0%) at levels comparable to prior classification methods without alterations. We find that machine learning classification from features extracted by a certified nonexpert crowd achieves high performance for ASD detection from natural home videos of the child at risk and maintains high sensitivity when privacy-preserving mechanisms are applied. These results suggest that privacy-safeguarded crowdsourced analysis of short home videos can help enable rapid and mobile machine-learning detection of developmental delays in children.

## Introduction

As digital and mobile healthcare becomes commonplace^1^, data captured by interactive mobile and wearable intervention systems^2–10^ result in video which can be used for continuous digital phenotyping^11–13^. The captured videos from these systems provide a rich data source which can be presented to humans who answer behavioral multiple choice questions about the video^14–16^, resulting in the video-wide annotation of behavioral features that are currently beyond the capabilities of automated methods. Incorporating human workers is crucial for annotating these behavioral features from video and audio samples, as the behaviors are too complex to be automatically measured. As mobile devices become increasingly pervasive, including in developing countries^17–19^, obtaining videos for a crowdsourced evaluation process can potentially accelerate early detection of developmental conditions for children who face geographic, economic, and social barriers to health care.

Crowdsourcing enables rapid human annotation of complex behavioral features in a scalable manner^20,21^. Because crowd workers can operate from anywhere in the world, diverse opinions can be aggregated into a consensus set of features, minimizing potential effects of noisy raters. However, low quality annotations can degrade the accuracy of the crowd’s prediction. In addition to low quality answers, different people have varying abilities to identify and discriminate social features of other people, let alone children. This extends to parents, who may be biased about how normal their child’s behaviors are in relation to other children. Optimized healthcare crowdsourcing workflows must therefore contain a certain level of selectivity in the workforce towards workers who can correctly identify abnormal deviations in subjective behavioral features such as social interaction quality, expressive language ability, and speech patterns.

A concern for crowdsourced video-based detection is data privacy, especially for a marginalized pediatric population recorded in the home setting. It is important to build trust with parents who want to receive an affordable and quick evaluation for their child but who may have apprehensions towards sharing video with strangers. Preserving the privacy contained in videos while maintaining enough information to provide a high-quality mobile detection tool is a critical challenge that must be addressed before digital detection tools, no matter how accurate and precise, can become actualized and widely adopted. Transparency and trust in digital health and AI solutions is crucial yet lacking, requiring innovation in trustworthy systems and methods^22,23^.

We test the hypothesis that a qualified (tested and trustable) crowd of non-expert workers recruited from paid platforms can efficiently tag features needed to run machine learning models for accurate detection of ASD, which is a complex neurodevelopmental disorder that impacts social, communication, and interest behaviors^24^. Some examples of ASD symptoms that cannot be detected with automated methods include ritualistic behaviors, narrow or extreme interests, resistance to change, difficulty expressing emotion, trouble following directions, minimal social responsiveness, and resisting physical contact^25–27^. We turn to humans to extract these complex behavioral features. Precisely quantifying the developmental phenotype is crucial for developing high-fidelity and accessible early diagnostic biomarkers for ASD^28–35^. Current diagnostic evaluations use behavioral instruments measuring dozens of behaviors in extended assessments^25,26^. While early detection leads to prompt intervention and better outcomes, the wait to receive formal assessments can surpass 1 year ^36^, and diagnosis is often delayed until children enter primary school^37,38^. This delay in diagnosis and subsequent treatment is more pronounced in underserved populations^39–41^. Data-driven approaches have estimated that over 80% of U.S. counties contain no ASD diagnostic resources^42^. The examinations must be administered in person by clinicians and take hours to complete^43–45^. As developmental conditions like ASD are dynamic and mutable phenotypes^46,47^, there remains an obligation to continuously monitor such conditions^48^. With rising developmental health concerns^49,50^, there is a need and opportunity for faster, scalable, and telemedical solutions.

Prior research has explored video-based diagnostic methodologies. Kanne et al. developed a mobile application where parents self-report answers to multiple choice questions about short video clips of their child^51^. The Systematic Observation of Red Flags (SORF) is a detection tool for ASD designed for observation of home videos of children^52^. Other efforts suggest that some crowd workers who are recruited on crowd platforms have the potential to provide high quality behavioral ratings^53–55^. The present study differs from these previous works in at least two ways: (1) we are the first study, to our knowledge, to fully crowdsource the task of providing human labels at scale for ASD detection or diagnostic purposes, and (2) we provide the first exploration of privacy-preserving mechanisms applied to the videos.

We demonstrate the potential of a distributed crowd workforce, selected through a multi-round virtual rater certification process, to accurately tag behavioral features of unstructured videos of children with ASD and matched controls between 1 and 7 years of age, both with and without privacy-preserving alterations to the video. We emphasize that we are testing the ability of workers recruited from the crowd to adequately and fairly score the features we care about without knowing about the underlying goal of ASD detection. Because the videos are short, evidence of several behavioral features we ask about do not appear in all videos. We ask workers to use their intuition about how the child would behave in reference to the question, and we hypothesize that these general impressions about a child from a short video clip could be useful behavioral features for diagnostic detection.

We feed the human-extracted behavioral features into two logistic regression ASD classifiers trained on score sheets from the ADOS^25^ observational instrument filled out by professional clinicians. The performances of the classifiers are used as a gold standard of crowd rater performance. We then evaluate the performances of the classifiers on a balanced set of 50 unstructured videos of children with ASD and matched controls. We evaluate median, mode, and mean aggregation methods of crowd responses for a single question, finding that the accuracy, precision, sensitivity, and specificity of each classifier are ≥ 95% across all metrics for the best aggregation strategy, outperforming all prior video-based detection efforts. We find that sensitivity (recall) of the classifiers is preserved, even with the most stringent privacy-preserving mechanisms.

These results suggest that privacy-preserved videos can potentially be used for remote detection of ASD. The benefit of leveraging the crowd for this task is in the feasibility of scaling up the presented process. This paper demonstrates that qualified crowd workers can be recruited to provide reliable behavioral annotations. In addition, we demonstrate the resilience and robustness of the technique against privacy-preserving video modifications.

## Materials and methods

All methods were carried out in accordance with relevant guidelines and regulations (Declaration of Helsinki). All experimental protocols were approved by the Stanford University Institutional Review Board (IRB) and the Stanford University Privacy Office. Informed consent was obtained from all subjects.

### Machine learning classifiers

**Figure 1:**
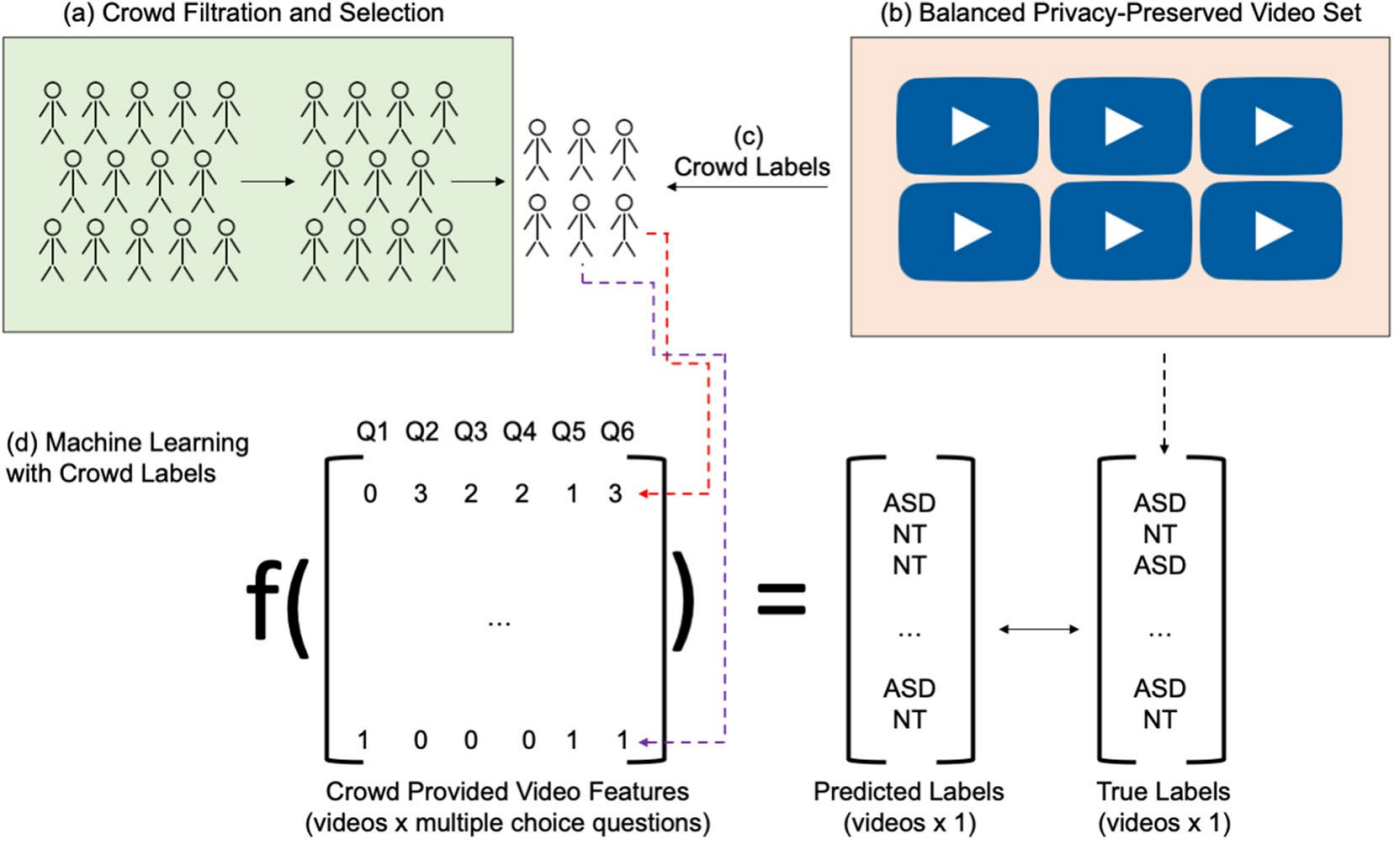
Overview of the crowd-powered AI detection process. (**a**) A trustworthy crowd is selected through a filtration process involving an evaluation set of videos. (**b**) A diagnosis and gender balanced set of unstructured videos are evaluated both with and without a set of privacy-preserving alterations: pitch shift and face obfuscation. (**c**) The curated crowd extracts behavioral features about the children in the videos by answering a set of multiple choice questions about the child’s behavior exhibited in the video, with each worker assigned to a random subset of the videos. (**d**) A classifier trained on electronic medical records (the “training set”) corresponding to the multiple choice answers to behavioral questions is used to predict the diagnosis from the aggregated video-wide annotations (the “test set”), and the classifications are compared against the known diagnoses in the video set (the “test set”).

Two previously validated^14,56^ binary ASD logistic regression classifiers were used to evaluate the quality of the crowd ratings (Fig. 1d). One classifier (LR5) was trained on archived medical records derived from the administration of the ADOS Module 2^25^ for 1,319 children with ASD and 70 non-ASD controls. We refer to this model as LR5 to indicate that it is a logistic regression classifier that has 5 input features. The other classifier (LR10) was trained on medical records from the ADOS Module 3^25^ for 2,870 children with ASD and 273 non-ASD controls. We refer to this model as LR10 to indicate that it is a logistic regression classifier that has 10 input features. As discussed in^34^, stepwise backward feature selection was applied to the ADOS electronic health record data to determine the top-5 and top-10 predictive features for ASD diagnosis in order to create a classifier with the minimal number of input features required for high performance. It is important to minimize the number of questions to avoid redundancy of questions and to lower the burden of crowd workers, which will result in higher detection throughput and greater scalability. The ADOS electronic health record data served as the “training set”; the aggregated crowd answers to multiple choice questions about public home videos served as the “test set”.

To calculate confidence intervals, we conducted permutation tests by bootstrapping the computations of AUROC, AUPRC, and point metrics for the unaltered conditions. We sampled with replacement new versions of the test set to evaluate against all metrics. We conducted 10,000 iterations and calculated the 95% confidence interval for each metric by sorting the resulting 10,000 metric values and recording the value at position 250 (2.5th percentile) and position 9,750 (97.5th percentile).

### Selection of videos

We recruited parents of children with ASD to share videos through advertising on social media and listservs. Parents were asked to upload the videos to YouTube or to share a link to a previously uploaded video. A representative collection (12 female ASD; 13 male ASD; 12 female neurotypical; 13 male neurotypical) of these videos and previously posted YouTube videos with sufficient descriptions of diagnosis, gender, and age was selected for both children with and without ASD (Fig. 1b). Videos for the ASD category were required to match the following criteria: (1) the child’s hand and face are visible, (2) opportunities for social engagement are present, and (3) an opportunity for using an object such as a toy or utensil is present. To curate a variety of videos, no further selection criteria were used. Of the 200 videos collected using this method, we selected a subset of 50 videos for the study. The selection of 50 videos used in the final study was based solely on child demographics. We randomly sampled female ASD, male ASD, female neurotypical, and male neurotypical videos to ensure either 12 or 13 children per category. We note that we did not filter or pre-select videos based on whether the videos exhibited the symptoms needed by our machine learning classifiers. For questions where the behavior in question was not exhibited in the video, we asked crowd workers to make their best guess about what the correct answer was. We call this method “*human imputation*.”

### Parent-reported diagnosis and clinician-provided severity levels

Diagnosis of the children in the videos was determined by parent-reported information or by video title and description reported by the uploader, e.g., “Joey with ASD at 36 months”. We also performed a *post hoc* analysis of the ASD severity level of the children represented in the videos by asking 7 licensed clinical experts who perform diagnostic evaluation for ASD as part of their job function to watch each video of the 25 children with an autism diagnosis and to rate the severity of the child’s autism symptoms according to the first question of the Clinical Global Impression (CGI) scale. The CGI measures the “severity of illness” between 1 (“normal, not at all ill”) to 7 (“among the most extremely ill patients”). We then recorded the mean rating rounded to the nearest whole number (Supplemental Figure S3). There were two videos with a mean rating of 2 (“borderline mentally ill”), two with a mean of 3 (“mildly ill”), four with a mean of 4 (“moderately ill”), eight with a mean of 5 (“markedly ill”), seven with a mean of 6 (“severely ill”), and two with a mean of 7 (“extremely ill”). We received at least 2 and up to 3 ratings per video. This was to validate that we posted a representative set of videos across the range of ASD severity levels. Because no children were rated as 1 (“normal, not at all ill”), we were able to provide evidence that a video-based evaluation of the children by clinicians was consistent with the parent-reported diagnoses.

### Recruitment of trustworthy and capable crowd workers

All experiments were conducted on Amazon Mechanical Turk (MTurk) (Fig. 1c). A different set of N=20 balanced public YouTube videos used in prior studies^16^ and selected as described above were used to filter crowd workers on MTurk from an initial pool of 1,107 workers to a set of 82 workers passing a set of quality control measures (Fig. 1a). To cast a wide net of potential crowd workers while maintaining some promise of quality, the initial pool was required to possess MTurk system qualifications indicating that they had completed at least 50 Human Intelligence Tasks (HITs) and had a cumulative approval rating above 80%. See Supplementary Information: Method S1 for a detailed description of the process. Crowd workers possessed no prior training or knowledge about the video rating task.

### Altering videos to achieve privacy conditions

We used established mechanisms to test both visual and audio privacy. To achieve visual privacy, we obfuscated the face with a red box, as illustrated in Supplemental Fig. S1. We used the *OpenCV* toolkit to draw boxes over the bounding box of the face as detected by a convolutional pretrained ResNet^57^ face detector. Frame smoothing was implemented to ensure that the face remained covered in the occasional frames where the face detector failed. In particular, when a face was not detected in the frame, the red box remained in the same position in all subsequent frames without a detected face until a new face position was detected. This ensured that a box was drawn near the child’s face throughout the duration of the video. To ensure perfect and complete coverage of the child’s face for all frames of the video, the processed videos were manually viewed and trimmed until complete face coverage was achieved.

To achieve audio privacy, we chose to use pitch shifting because it preserves all of the original content of the speech while obfuscating potentially identifying vocal features. We used *ffmpeg* to extract the audio from the original video, pitch shift the audio down by a factor of 10/7, then append the new audio clip to a new video constructed from the sequential JPG frames of the original video.

## Results

### Crowdsourced behavioral feature extraction from video

We constructed a formal pipeline to aggregate a steady-state population of trustworthy and competent workers from a broad crowd whose answers to behavioral questions about videos would yield high detection performance when fed as input into a machine learning classifier. Prior work has demonstrated that features extracted by non-expert raters can yield high diagnostic performance on unstructured videos^14^. Yet, no prior literature to our knowledge has demonstrated the capacity of crowdsourced ratings for diagnosis or detection of developmental conditions. We created a series of Human Intelligence Tasks (HITs) on the Amazon Mechanical Turk (MTurk) crowdsourcing platform to recruit crowd workers (see “Recruitment of trustworthy and capable crowd workers” for details). We initially evaluated 1107 randomly selected crowd workers through our virtual rater certification process and filtered the crowd down to 102 consistently high-performing workers who provided complete feature vectors with consistent results.

To extract categorical ordinal behavioral features for each video, we published a HIT for each of a balanced set of 50 unstructured videos of children (25 ASD, 25 neurotypical; 26 male, 24 female). Each HIT contained the embedded video of the child with a potential developmental condition and a series of 31 multiple choice behavioral questions (see Supplemental Information Fig. S1 for a visualization of the interface and Supplemental Information File S1 for the full list of behavioral questions asked in each HIT). Due to the low number of behavioral features used as input to the classifiers (see Supplementary Information S1: Machine learning classifiers for details), we did not provide raters with the opportunity to answer “N/A” to a particular question. We instead requested for raters to predict what the behavior for the child would be using their intuition. Workers were not told that their task was to provide answers for diagnosis or detection ASD, and they were not informed about the purpose of their multiple choice answers.

We hypothesized that some crowd workers would exhibit a high level of intuition about certain ASD-related behaviors given other behaviors. While we only used a subset of the 31 questions as inputs to the classifiers, the unused questions served as quality control opportunities (see “Recruitment of trustworthy and capable crowd workers” for details). We randomly sampled 3 crowd workers from the filtered crowd to perform each HIT. Three workers were chosen per condition based on prior experiments by Tariq et al. demonstrating that 3 human raters are sufficient for the classifier performance to converge^14^.

The importance of trust in healthcare solutions, especially with machine learning approaches deployed on mobile devices, cannot be overstated. We explored the effect of privacy-preserving mechanisms in the visual and audio domains on classifier performance. We published an identical set of HITs with 3 privacy-preserving mechanisms: (1) full obfuscation of the face with a red face box (visual privacy; see Supplemental Information Fig. S1), (2) pitch shifting the audio of the child to a lower frequency (audio privacy), and (3) a combination of both approaches (visual and audio privacy). As with the unaltered video tasks, we randomly sampled 3 crowd workers from the filtered crowd to perform each HIT.

### Performance of ASD classifiers

We evaluated the quality of the crowd’s answers using two logistic regression classifiers trained on scoresheets generated from the use of the ADOS observational instrument. We used one logistic regression classifier (which we call LR5 for brevity) trained on 5 highly predictive questions from the ADOS and another classifier (which we call LR10 for brevity) trained on a different set of 10 highly predictive questions from the ADOS. We plotted the Receiver Operating curves (ROC) for all conditions, where the true positive rate is plotted against the false positive rate for different class cutoffs of the logistic regression classifier’s output probability. We also plotted Precision-Recall curves (PRC), where precision is plotted against recall for different class cutoffs. We measured the Area Under the Receiver Operating Characteristic curve (AUROC) and the Area Under the Precision-Recall curve (AUPRC) for all classifiers and conditions. For both of these metrics, values closer to 1.0 indicate better performance (1.0 means perfect classification) while values closer to 0.5 indicate random guessing by the classifier.

**Figure 2:**
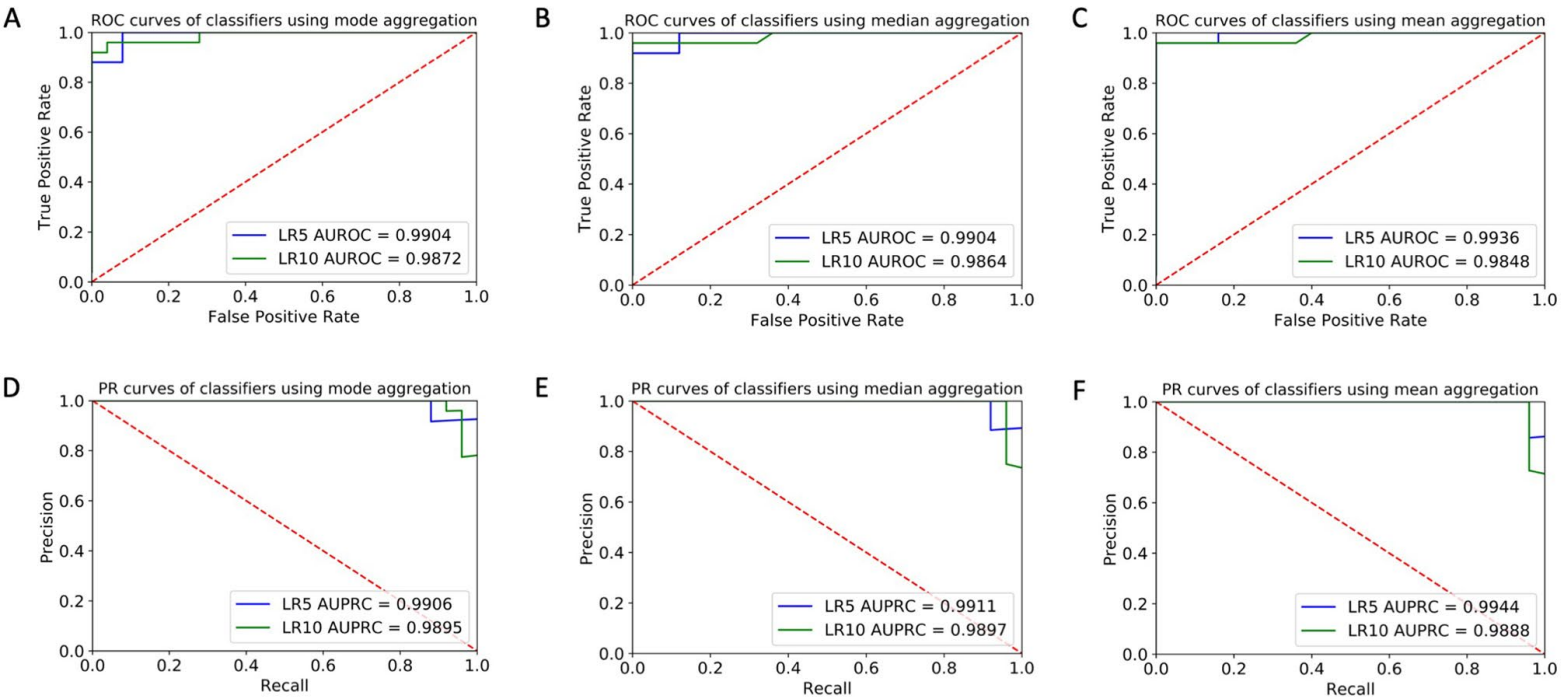
Receiver Operating Characteristic (ROC) and Precision-Recall (PR) curves of the classifiers trained on aggregated features from the filtered crowd raters. The blue line shows the performance of the LR5 classifier and the green line shows the performance of the LR10 classifier. ROC curves for input features to the classifier are aggregated using the (**A**) mode, (**B**) round of the mean, and (**C**) median of the crowd worker responses. The true positive rate is plotted against the false positive rate for different class cutoffs of the logistic regression classifier’s output probability. PR curves for input features to the classifier are aggregated using the (**D**) mode, (**E**) round of the mean, and (**F**) median of the crowd worker responses. Precision is plotted against recall for different class cutoffs of the logistic regression classifier’s output probability. For both ROC and PR curves, area under the curves increasingly closer to 1.0 indicate increasingly better performance, and a value of 0.5 indicates random guessing by the classifier.

We first aggregated the 3 crowd sourced responses for each video by taking the mode of the answers to each question, breaking ties randomly. The mode of each crowd worker response was used as the input to the classifiers. The AUROC of the LR10 classifier was 0.9872 ± 0.02 while the AUROC of the LR5 classifier was 0.9904 ± 0.02 with mode aggregation (Fig. 2a). The AUPRC of the LR10 classifier was 0.9895 ± 0.02 while the AUPRC of the LR5 classifier was 0.9906 ± 0.02 with mode aggregation (Fig. 2d). The LR10 classifier achieved 96.0% ± 5.0% accuracy, 100.0% ± 0.0% precision, 92.0% ± 10.0% sensitivity / recall, and 100.0% ± 0.0% specificity (Table 1), and the LR5 classifier achieved 92.0% ± 7.0% accuracy, 95.7% ± 7.0% precision, 88.0% ± 13.0% sensitivity / recall, and 96.0% ± 6.7% specificity with mode aggregation (Table 1).

**Table 1:** Performance of the machine learning classifiers on aggregated crowd features when using the majority rules (mode), median, and mean aggregation methods.

	Accuracy (%)		Precision (%)		Sensitivity/recall (%)		Specificity (%)	
	LR10	LR5	LR10	LR5	LR10	LR5	LR10	LR5
Mode	96.0 ± 5.0	92.0 ± 7.0	100.0 ± 0.0	95.7 ± 7.0	92.0 ± 10.0	88.0 ± 13.0	100.0 ± 0.0	96.0 ± 6.7
Median	92.0 ± 7.0	92.0 ± 7.0	88.9 ± 12.0	92.0 ± 10.4	96.0 ± 6.8	92.0 ± 10.0	88.0 ± 13.0	92.0 ± 10.4
Mean (rounded)	90.0 ± 8.0	98.0 ± 3.0	85.7 ± 12.4	100.0 ± 0.0	96.0 ± 6.8	96.0 ± 6.8	84.0 ± 13.7	100.0 ± 0.0

Performance metrics from the LR10 and LR5 classifiers are shown respectively. A probability threshold of 0.5 was used to distinguish the ASD and neurotypical classes.

We next aggregated the crowdsourced responses by using the median response of crowd workers as the input to the classifiers. The AUROC of the LR10 classifier was 0.9864 ± 0.02 while the AUROC of the LR5 classifier was 0.9904 ± 0.02 with median aggregation (Fig. 2b). The AUPRC of the LR10 classifier was 0.9897 ± 0.02 while the AUPRC of the LR5 classifier was 0.9911 ± 0.02 with median aggregation (Fig. 2e). The LR10 classifier achieved 92.0% ± 7.0% accuracy, 88.9% ± 12.0% precision, 96.0% ± 6.8% sensitivity / recall, and 88.0% ± 13.0% specificity (Table 1), and the LR5 classifier achieved 92.0% ± 7.0% accuracy, 92.0% ± 10.4% precision, 92.0% ± 10.0% sensitivity / recall, and 92.0% ± 10.4% specificity with median aggregation (Table 1).

Finally, we aggregated the crowdsourced responses by taking the mean of the categorical ordinal variables and rounding the answer to the nearest whole number. The AUROC of the LR10 classifier was 0.9848 ± 0.03 while the AUROC of the LR5 classifier was 0.9936 ± 0.01 with mean aggregation (Fig. 2c). The AUPRC of the LR10 classifier was 0.9888 ± 0.02 while the AUPRC of the LR5 classifier was 0.9944 ± 0.01 with mean aggregation (Fig. 2f). The LR10 classifier achieved 90.0% ± 8.0% accuracy, 85.7% ± 12.4% precision, 96.0% ± 6.8% sensitivity / recall, and 84.0% ± 13.7% specificity (Table 1), while the LR5 classifier achieved 98.0% ± 3.0% accuracy, 100.0% ± 0.0% precision, 96.0% ± 6.8% sensitivity / recall, and 100.0% ± 0.0% specificity with mean aggregation (Table 1).

### Performance using privacy-preserving mechanisms

We studied the effect of privacy-preserving mechanisms on the performance of the crowd. We evaluated the performance of MTurk workers on the same balanced set of 50 videos with all faces obfuscated, with audio pitch shifted down, and with both faces obfuscated and audio pitch shifted. Each worker was assigned to one privacy condition per video. This allowed us to quantify the effects of visual and audio privacy mechanisms on non-expert ratings.

**Figure 3:**
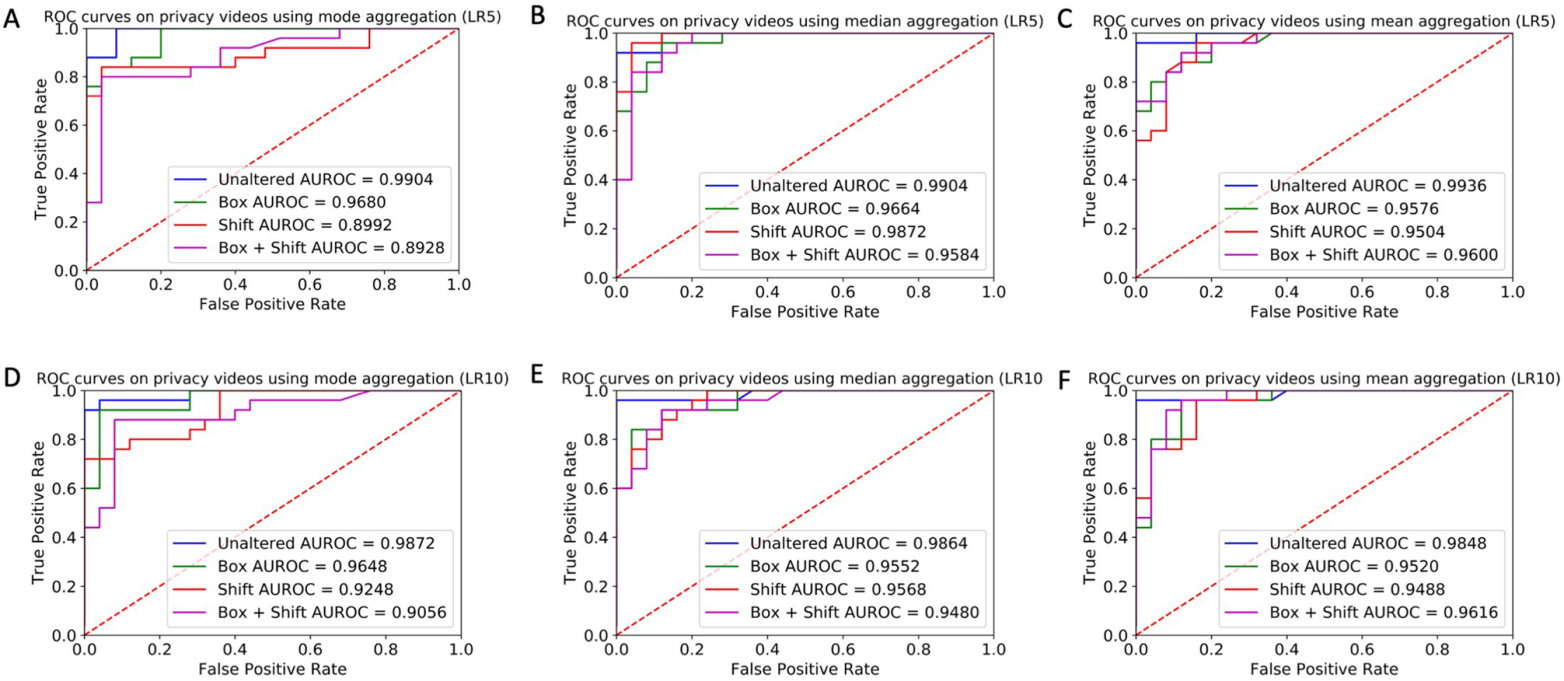
ROC curves of the classifiers trained on aggregated features from the filtered crowd raters under each privacy condition. The true positive rate is plotted against the false positive rate for different class cutoffs of the logistic regression classifier’s output probability. The color of the curve represents the privacy condition: blue represents unaltered videos, green represents face obfuscation, red represents pitch shift, and purple represents face obfuscation and pitch shift. Plots show aggregated results using the (**A**,**D**) mode, (**B**,**E**) median, and (**C**,**F**) round of the mean of the crowd worker responses. The ROC curves are shown for both the LR5 (**A**–**C**) and LR10 (**D**–**F**) classifiers. Area under the curves increasingly closer to 1.0 indicate increasingly better performance, and a value of 0.5 indicates random guessing by the classifier.

The lowest AUROC for any aggregation method, classifier, and privacy condition was 0.8928 ± 0.09, using the mode aggregation strategy (Fig. 3a). By contrast, the lowest median aggregation AUROC was 0.9480 ± 0.06 (Fig. 3e) and the lowest mean aggregation AUROC was 0.9488 ± 0.05 (Fig. 3). Using all three aggregation methods, all privacy conditions lowered the AUROC of both the LR5 and LR10 classifiers compared to the baseline unaltered condition (Fig. 3). The robustness of the ROC curve against privacy alterations appears to vary across aggregation strategies. While the unaltered ROC curves are nearly identical for the unaltered conditions regardless of aggregation strategy used (Fig. 2), the privacy conditions introduce variance in curve shape and AUROC values across privacy mechanisms, highlighting the importance of the aggregation strategy chosen.

**Figure 4:**
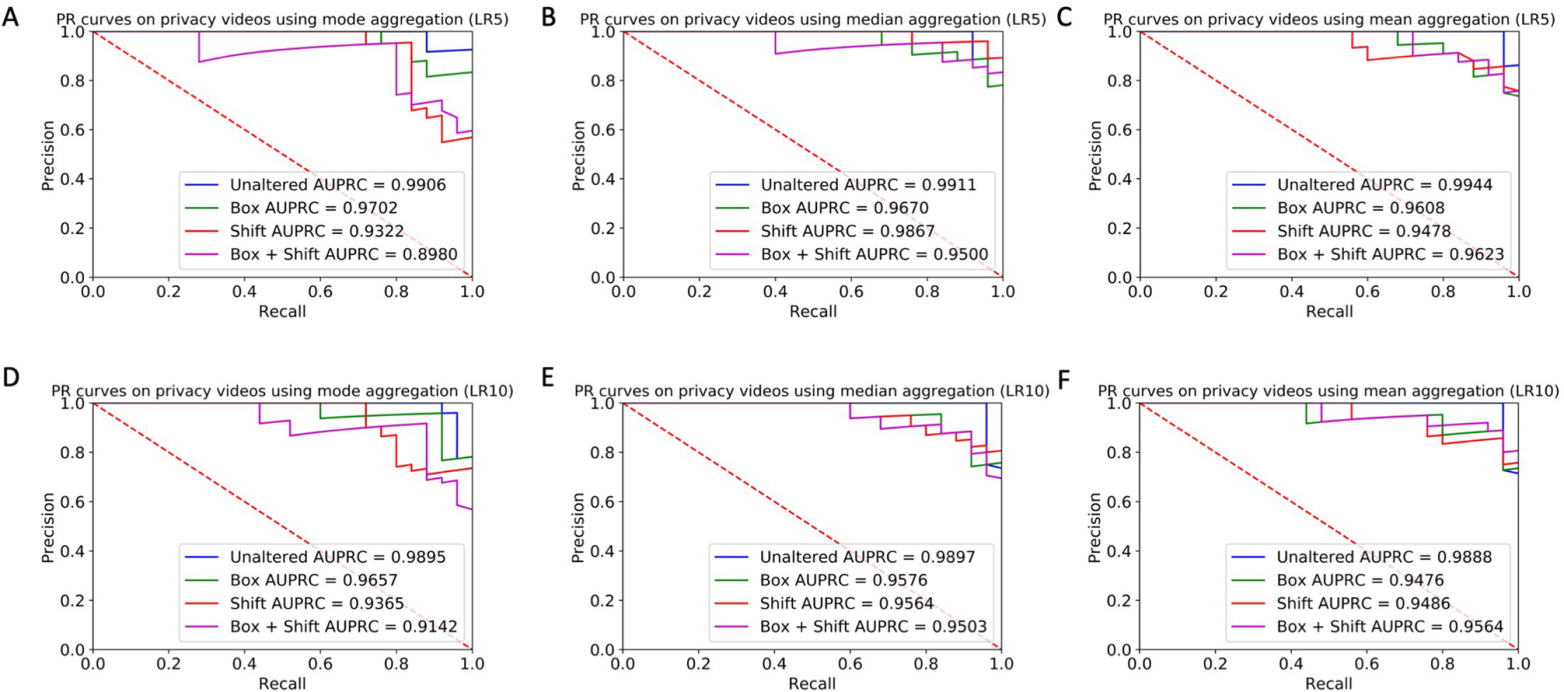
PR curves of the classifiers trained on aggregated features from the filtered crowd raters under each privacy condition. Precision is plotted against recall for different class cutoffs of the logistic regression classifier’s output probability. The color of the curve represents the privacy condition: blue represents unaltered videos, green represents face obfuscation, red represents pitch shift, and purple represents face obfuscation and pitch shift. Plots show aggregated results using the (**A**,**D**) mode, (**B**,**E**) median, and (**C**,**F**) round of the mean of the crowd worker responses. The ROC curves are shown for both the LR5 (**A**–**C**) and LR10 (**D**–**F**) classifiers. Area under the curves increasingly closer to 1.0 indicate increasingly better performance, and a value of 0.5 indicates random guessing by the classifier.

The lowest AUPRC for any aggregation method, classifier, and privacy condition was 0.8980 ± 0.09 (Fig. 4) for the mode aggregation strategy. The relative effects of the privacy conditions on AUPRC were nearly identical to the effects on AUROC (Fig. 3). All privacy conditions lowered the AUPRC with respect to the baseline unaltered condition (Fig. 4). Like with AUROC, the PR curves varied across aggregation strategies (Fig. 4), with the lowest AUPRC for mode aggregation (0.8980 ± 0.10; Fig. 4a) manifesting noticeably lower than the lowest AUPRC under any privacy condition for both median (0.9500 ± 0.07; Fig. 4b) and mean (0.9476 ± 0.07; Fig. 4f) aggregation strategies.

Mean and median crowd worker aggregation strategies appear more robust to privacy-altering modifications than the majority-rules (mode) strategy in terms of both AUROC (Fig. 3) and AUPRC (Fig. 4). This effect is likely due to the cumulative effect of multiple cases where there were no consensus answers between crowd raters on an individual question. In particular, there were 69 (video, question) pairs (out of a total 390 possibilities) where there was not a consensus category chosen by the 3 raters, 64 pairs in the face box conditions, 96 pairs in the pitch shift condition, and 125 pairs in the combined case.

When using the median and mean aggregation methods, the sensitivity (recall) of both the LR5 and LR10 classifiers was not degraded with any privacy condition, regardless of the classifier used (Tables 2 and 3). This protective effect against sensitivity was not present with mode aggregation. With the LR10 classifier, the accuracy, precision, and specificity from any privacy condition was lower than or equal to the unaltered condition using all aggregation methods (Table 2), except that the face box resulted in higher specificity when using mean aggregation. With the LR5 classifier, the accuracy, precision, and specificity from any privacy condition was lower than or equal to the unaltered condition using all aggregation methods (Table 3). There is no clear difference in the face box and pitch privacy mechanisms in terms of severity of classifier performance degradation; the effect is dependent on the aggregation methods used. Dramatic differences in classifier performance using different aggregation methods but with all else held equal appeared in several instances: the largest differences across aggregation strategies for LR10 were 12.0% for accuracy (face box; mode vs. mean aggregation), 22.3% for precision (face box; mode vs. mean aggregation), 20.0% for sensitivity (pitch shift; mode vs. median and mean aggregations), and 24.0% for specificity (pitch shift and combined conditions; mode vs. mean aggregations) (Table 2). The largest differences for LR5 were 12.0% for accuracy (combined condition; mode vs. mean aggregation), 9.3% for precision (combined condition; median vs. mean aggregation), 20.0% for sensitivity (combined condition; mode vs. median aggregation), and 16.0% for specificity (combined condition; median vs. mean aggregation) (Table 3).

**Table 2:** Performance of the LR10 classifier on aggregated crowd features across privacy-preserving mechanisms when using the mode, median, and mean aggregation methods, respectively.

Privacy mechanism	Accuracy (%)			Precision (%)			Sensitivity [Recall] (%)			Specificity (%)		
	Mode	Median	Mean	Mode	Median	Mean	Mode	Median	Mean	Mode	Median	Mean
Unaltered	96.0	92.0	90.0	100.0	88	85.7	92.0	96.0	96.0	100.0	88.0	84.0
Face box	94.0	88.0	82.0	95.8	85.2	73.5	92.0	92.0	100.0	96.0	84.0	96.0
Pitch shift	82.0	82.0	88.0	83.3	73.6	71.4	80.0	100.0	100.0	84.0	64.0	60.0
Face box and pitch shift	86.0	78.0	80.0	84.6	70.1	71.4	88.0	96.0	100.0	84.0	60.0	60.0

Sensitivity of the classifier is retained even with the most stringent privacy-preserving mechanisms. A probability threshold of 0.5 was used to distinguish the ASD and neurotypical classes.

**Table 3:** Performance of the LR5 classifier on aggregated crowd features across privacy-preserving mechanisms when using the mode, median, and mean aggregation methods, respectively.

Privacy mechanism	Accuracy (%)			Precision (%)			Sensitivity [Recall] (%)			Specificity (%)		
	Mode	Median	Mean	Mode	Median	Mean	Mode	Median	Mean	Mode	Median	Mean
Unaltered	92.0	92.0	98.0	95.7	92.0	100.0	88.0	92.0	96.0	96.0	92.0	100.0
Face box	86.0	88.0	86.0	87.5	82.8	80.0	84.0	96.0	96.0	88.0	80.0	76.0
Pitch shift	84.0	92.0	88.0	84.0	86.2	82.8	84.0	100.0	96.0	84.0	84.0	80.0
Face box and pitch shift	76.0	82.0	88.0	74.1	73.5	82.8	80.0	100.0	96.0	72.0	64.0	80.0

Sensitivity of the classifier is retained even with the most stringent privacy-preserving mechanisms. A probability threshold of 0.5 was used to distinguish the ASD and neurotypical classes.

## Discussion

Our results confirm the hypothesis that a qualified crowd of non-expert workers from paid platforms can efficiently tag features needed to run machine learning models for accurate detection of ASD. We emphasize that we are testing the ability of workers recruited from the crowd to adequately and fairly score the features we care about without knowing anything about the underlying detection task. We are able to derive accurate diagnoses through feeding the crowd workers’ responses into machine learning classifiers.

This is the first *crowdsourced* study of human-in-the-loop machine learning methods for detection of any behavioral condition, focusing on pediatric ASD as a challenging case study. When aggregating the categorical ordinal behavioral features provided by the crowd, the best classifier using the optimal aggregation strategy for this dataset (mean) yielded ≥ 96% performance for accuracy, precision, sensitivity (recall), and specificity. This performance exceeds alternative classification methods that do not employ crowdsourcing, with notable prior results achieving an accuracy of 88.9%, sensitivity of 94.5%, and specificity of 77.4% on the best-performing classifier^14^ on a different video dataset. This suggests that when privacy-preserving mechanisms are not applied to videos, the methods described here can still work. However, we emphasize that larger studies with a fully representative cohort are required before the solution described here can be translated into clinical settings.

Even with privacy mechanisms in place, the results perform slightly higher than AI-based video phenotyping of ASD absent of crowdsourcing and privacy protection^14^. The LR5 classifier, used on crowd responses to videos with both pitch shift and face obfuscation applied, still achieved 88.0% accuracy, 96.0% sensitivity, and 80.0% specificity using mean aggregation. These results are comparable to the unaltered video classifiers in prior work^14^. Because the sensitivity was preserved, the method can potentially provide privacy-preserved detection for ASD in a scalable and accessible manner.

While this work does not constitute a clinical study, we are interested in how the proposed methods can eventually be leveraged in diagnostic practices. One potential use case could be the integration of the methods described here with commercial telehealth solutions for pediatric behavioral diagnostics, where behavioral measures are needed but can be rate-limited by the number of coders. Such tools can aid clinicians in finding children who have an increased risk of ASD, helping to speed up the currently long waitlists^36^ for starting and receiving care. However, before such translational use cases can be realized and implemented in a health care system, larger studies, including official clinical trials, will be required to fully evaluate the potential of the presented methods to translate to clinical practice. We believe that digital health care solutions in general, including approaches like ours, will allow for more effective detection and diagnosis of behavioral, mental, and developmental health conditions.

In an age where privacy of personal data is at the forefront of geopolitical issues and public discourse, trust is paramount for effective data sharing. We found that those parents who would not share raw videos of their children would share the videos after our privacy-preserving steps were applied. Importantly, applying these mechanisms to the videos did not degrade the sensitivity of the classifier but did degrade the specificity. More work on trustworthy AI will be needed to maximize both trust and the utility of data being shared^57^.

We propose and implement a structured process of (1) applying feature selection on electronic medical record data to determine the behaviors most predictive of a particular condition, (2) training machine learning classifiers to predict a diagnosis with the minimal feature set on the electronic medical record data, (3) building a diagnostically and demographically balanced training library of videos enriched for those features, (4) applying privacy transformations to those videos, (5) recruiting a curated crowd workforce, (6) assigning members of the curated crowd to behaviorally tag subsets of the videos, and finally, (7) providing a diagnosis by feeding in the aggregated crowd responses as input to the machine learning classifier. This process can likely be applied to other developmental conditions, enabling scalable telemedical practices. In order for the presented technique to truly scale, manual annotators must check the quality of the privacy modifications. To preserve privacy during these manual checks, the image can be transformed into a feature representation that maintains the human outline while preserving privacy, such as dense optical flow. As object detection methods, and in particular face detection, improve, we expect this human requirement to lessen. In the meantime, however, the task of manually checking the privacy alterations can be crowdsourced without the need for curated workers, enabling scalability of the overall approach.

This work is the first published case, to our knowledge, of “*human imputation*”, where humans can fill in the missing data in the questionnaires using their intuition about the child. All videos were short, ranging from 15 to 129 s (mean = 42.4 s; SD = 24.9 s), and sometimes only illustrating a few of the behavioral features used in the classifiers. Nevertheless, raters were capable of rating the missing behaviors to a sufficient degree to realize strong classifier performance. Because clinicians may be unwilling to answer questions about unobserved behavior, this methodology could prove promising when incomplete data are available for a patient, which is often the case in longitudinal at-home data monitoring efforts. We note that while this methodology may not suffice for a formal diagnosis, it may help to increase the throughput and scalability of remote detection efforts.

There are several limitations of the present study. An important limitation is that some of the questions the crowd workers were asked, while not used as input to either the LR5 or LR10 classifiers, did mention autism in the wording, potentially biasing their inputs in the direction of increased severity of symptoms. We conducted an analysis of worker response distributions for each question across videos to verify whether this was the case (Supplemental Figure S2) and found no noticeable answer biases towards either more severe or less severe symptom ratings. In an ideal setting, workers would not have received any communication about “autism” and would simply annotate observed behaviors in the video.

While the dataset used was balanced for gender and diagnosis, the unstructured nature of the videos could introduce uncontrolled confounders. Diagnosis was based on self-reporting from parents, introducing the potential for discrepancies in the diagnostic reports of the child. ASD is a heterogeneous spectrum condition, and the phenotype is not binary. However, the analysis performed in the present study treats ASD as a binary condition, not capturing subtleties in children who may “almost have ASD” or “barely have ASD”. One possible approach for future work would involve using the probabilities emitted from the logistic regression classifier as an estimate of ASD severity. Another limitation introduced by this study design is the inability to attribute the degradation of performance to either a lack of ability of workers to “impute” the missing video data or the natural degradations that would result from the privacy-preserving mechanisms. Future work evaluating the granular effects of video-based privacy techniques on item-level answers would result in greater translatability of the results to the clinic. A final and crucial limitation is that while the videos we selected were balanced by age, gender, and diagnosis, there are undoubtably multiple biases in the selected video sample, requiring further work on larger samples to evaluate how the discussed methods will scale for all populations.

## Conclusion

Crowd-powered machine learning methods for detection of developmental delays, such as the general pipeline illustrated here, address needs in translational and computational psychiatry, fields currently embracing scalable and accessible solutions^58^. Increasingly, it is crucial that these solutions maintain user trust^59^, especially if deployed in home settings with protected populations such as children with ASD. Machine learning solutions alone, without incorporating human insight, are far from providing precise developmental diagnostics at the level of a professional psychiatrist. We demonstrate the first crowdsourced study of human-in-the-loop machine learning methods for detection of ASD in a privacy-preserved manner. We find that when drawing a large but capable subset of the crowd filtered using a short series of worker evaluation tasks, the filtered crowd workers can be sampled to answer behavioral multiple-choice questions about unstructured videos of children with potential developmental conditions. Even with privacy mechanisms in place, the results reported here slightly outperform the best performance of video-based ASD detection by nonexperts reported in prior literature. Crowd-powered and privacy-preserved detection systems such as the one described here have the potential to inspire scalable and accessible solutions to pediatric healthcare.

## Supplementary Information

Supplementary Information.
