# Supplementary material for: Crowdsourced privacy-preserved feature tagging of short home videos for machine learning ASD detection
Source: Sci Rep. 2021 Apr 7;11:7620. doi: 10.1038/s41598-021-87059-4 (PMC8027393; doi:10.1038/s41598-021-87059-4)
Supplement: Supplementary file 1 — Supplementary Information. [file 41598_2021_87059_MOESM1_ESM.docx]

**Supplementary Information**

**Title:** Crowdsourced privacy-preserved feature tagging of short home videos for machine learning ASD detection

**Authors:**

Peter Washington^1^, Qandeel Tariq^2^, Emilie Leblanc^3^, Brianna Chrisman^1^, Kaitlyn Dunlap^3^, Aaron Kline^3^, Haik Kalantarian^3^, Yordan Penev^3^, Kelley Paskov^4^, Catalin Voss^5^, Nathaniel Stockham^6^, Maya Varma^5^, Arman Husic^3^, Jack Kent^3^, Nick Haber^7^, Terry Winograd^5^, Dennis P. Wall^3,4,8^*

**Affiliations**:

^1^Department of Bioengineering, Stanford University, Stanford, California, USA.

^2^Research Scientist, Amazon, Seattle, Washington, USA.

^3^Department of Pediatrics (Systems Medicine), Stanford University, Stanford, California, USA.

^4^Department of Biomedical Data Science, Stanford University, Stanford, California, USA.

^5^Department of Computer Science, Stanford University, Stanford, California, USA.

^6^Department of Neuroscience, Stanford University, Stanford, California, USA.

^7^Graduate School of Education, Stanford University, Stanford, California, USA.

^8^Department of Psychiatry and Behavioral Sciences (by courtesy), Stanford University, Stanford, California, USA.

*To whom correspondence should be addressed: dpwall@stanford.edu


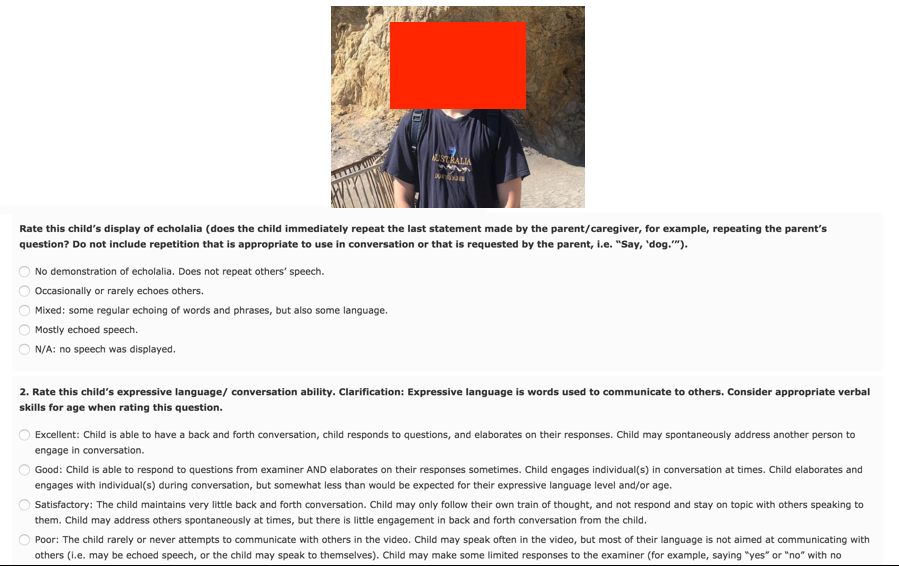


**Supplementary Fig. S1.** **Interface of the clinical-grade video phenotyping crowdsourced task.** A video is displayed on the top of the page followed by a series of multiple-choice questions about the video. The answers to these questions serve as categorical ordinal variables which can be used as features to a machine learning classifier.


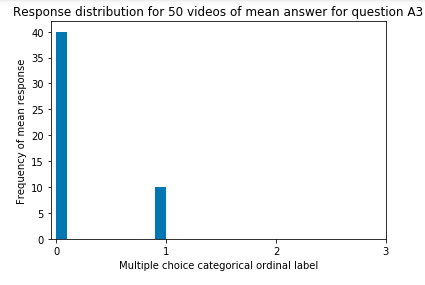

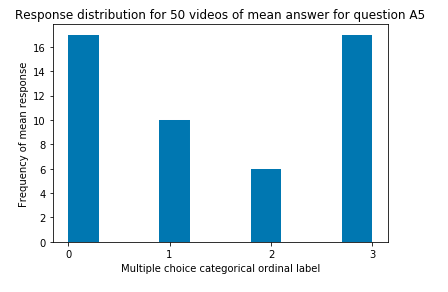

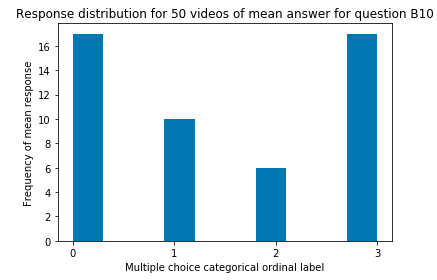


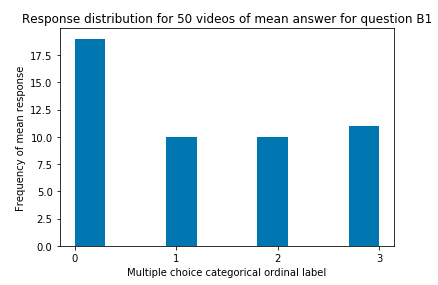

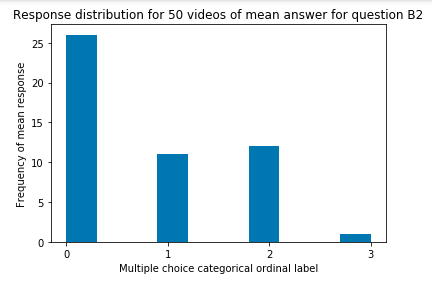

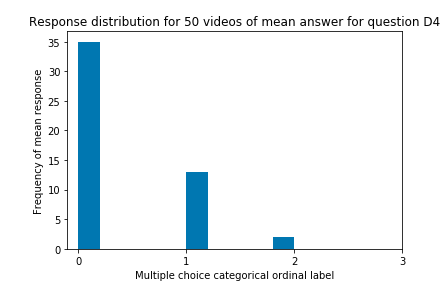

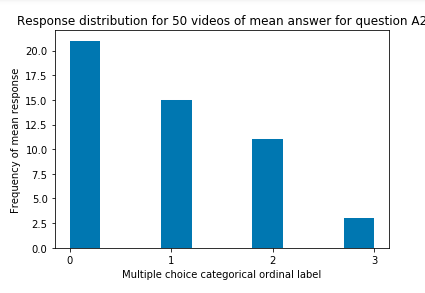

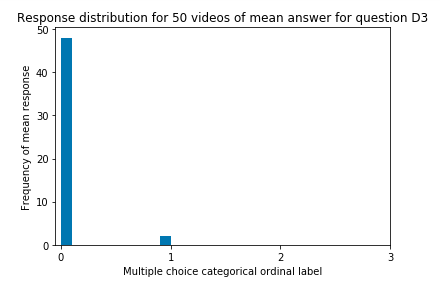

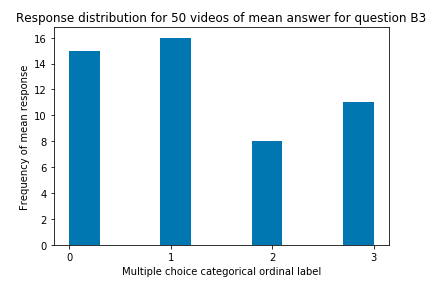

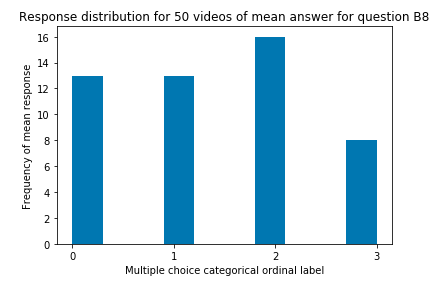

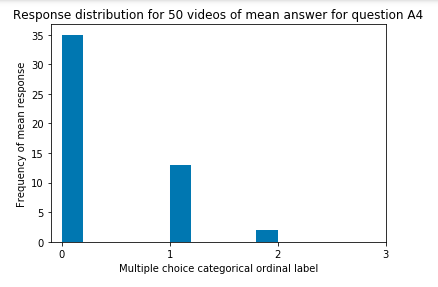

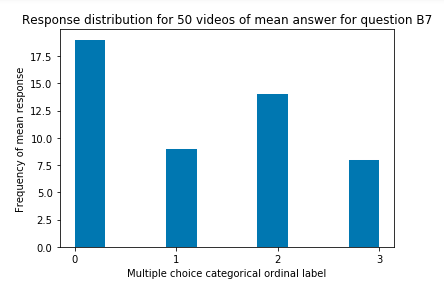


**Supplementary Fig. S2. Worker response distribution for all questions across all 50 videos.** A3 corresponds to question 1; A5 to question 2; B1 to question 8; B2 to question 4; A2 to question 3; D3 to question 5; B3 to question 15; B8 to question 19; A4 to question 25; B7 to question 28, and D4 to question 30.


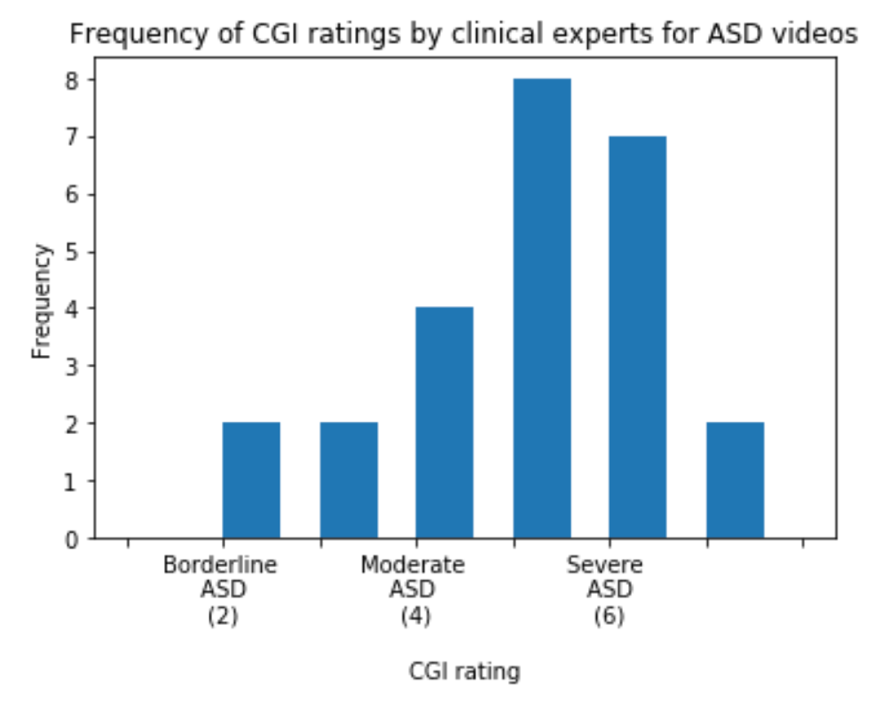


**Supplementary Fig. S3. Distribution of mean Clinical Global Impression (CGI) ratings by professional clinicians for the video dataset used.**

|  | **Unaltered** | **Face Box** | **Pitch Shift** | **Box and Shift** |
| --- | --- | --- | --- | --- |
| Allowed: The video would help research and other people. | 143 | 87 | 80 | 59 |
| Allowed: There is no reason to hide the contents of the video. | 97 | 41 | 38 | 24 |
| Allowed: The addition of the privacy mechanism makes the video amenable to sharing. | N/A | 198 | 13 | 174 |
|  |  |  |  |  |
| Not allowed: There are privacy concerns with sharing the video. | 168 | 60 | 185 | 47 |
| Not allowed: The content present in the video is inappropriate for public sharing. | 78 | 48 | 37 | 31 |
| Not allowed: The privacy mechanism removes the usefulness of the video for research. | N/A | 30 | 38 | 63 |
| Not allowed: The privacy mechanism makes the video no longer desirable for public sharing. | N/A | 29 | 105 | 85 |
| Not allowed: The video is not related to research. | 11 | 7 | 6 | 8 |

**Supplemental Table S1.** **Results of the survey of MTurk parents regarding permissions for sharing video under various privacy-preserving alterations**. Justifications provided by parents regarding their survey responses for each condition were categorized by two authors independently, with disagreements between researchers settled by a third rater. Raters with explanations that cannot be categorized due to insufficient explanation are not included in the table.

# Rate this child’s display of echolalia (does the child immediately repeat the last statement made by the parent/caregiver, for example, repeating the parent’s question? Do not include repetition that is appropriate to use in conversation or that is requested by the parent, i.e. “Say, ‘dog.’”).

- (0) No demonstration of echolalia. Does not repeat others’ speech.
- (1) Occasionally or rarely echoes others.
- (2) Mixed: some regular echoing of words and phrases, but also some language.
- (3) Mostly echoed speech

# Rate this child’s expressive language/ conversation ability:

*Clarification: Expressive language is words used to communicate to others. Consider appropriate verbal skills for age when rating this question.*

- (0) Excellent: Child is able to have a back and forth conversation, child responds to questions, and elaborates on their responses. Child may spontaneously address another person to engage in conversation.
- (1) Good: Child is able to respond to questions from examiner AND elaborates on their responses sometimes. Child engages individual(s) in conversation at times. Child elaborates and engages with individual(s) during conversation, but somewhat less than would be expected for their expressive language level and/or age.
- (2) Satisfactory: The child maintains very little back and forth conversation. Child may only talk about things they are interested in, and not respond and stay on topic with others speaking to them. Child may address others spontaneously, but there is little engagement in back and forth conversation from the child.
- (3) Poor: The child rarely or never attempts to communicate with others in the video. Child may speak often in the video, but most of their language is not aimed at communicating with others (i.e. may be echoed speech, or the child may speak to themselves). Child may make some limited responses to the examiner (for example, saying “yes” or “no” with no elaboration).

# Rate this child’s speech patterns.

*Clarification: Abnormal speech includes: a*bnormal volume, pitch, intonation, rate, rhythm, stress, prosody or volume in speech. Consider appropriate language abilities for the child's age while rating.

- (0) Child has no abnormal qualities to speech and has appropriate volume, intonation, rhythm, rate, pitch, etc. when speaking.

o (1) Child has slightly unusual qualities to speech. For example, speaking too softly or too loudly, monotone or exaggerated intonation at times, speech that is too fast or too slow.

- (2) Child’s speech is abnormal. The child’s speech patterns make them hard to understand, due to odd volume, tone, rate/rhythm, intonation. Speech could be extremely monotone, pitch that seems unnatural (too high or too low), rhythm and pitch that seems robotic, or overly animated.
- (3) Stutter or stammer, and probable speech delay/disorder.

# Rate this child’s change in gaze, facial expression, and gesture while the child vocalizes. Include vocalizations used to maintain interaction or to respond to a person, as well as initiations

- (0) Child’s vocalization is paired with appropriate facial expression, gesture, gaze.
- (1) Child’s vocalization is paired with abnormal, limited frequency and/or range of gesture, gaze and facial expression. Child may only rely on only gesture, or only gaze, or only vocalization.
- (2) Child rarely or never links their vocalization with nonverbal communication, i.e. no or minimal use of gestures, facial expression, or socially directed gaze.
- (3) Child may avoid direct gaze, but could be attributed to shyness. Shows some use of nonverbal communication linked with vocalization.

# Rate the child’s self-injurious or aggressive behaviors towards themselves. This includes behaviors such as biting his/herself, hitting his/her head, or pulling his/her hair.

- (0) Does not harm him/herself.
- (1) Unclear. Some possible self-injury, and/or very rare self-injury.
- (2) At least one clear example of self-injury.

# Does the child entertain [himself/herself] or find things to do without others help?

- (0) Yes, always entertains him/herself.
- (1) Often does this.
- (2) Sometimes does this.
- (3) Rarely or never entertains him/herself.

# Does the child understand spoken language

- (0) Yes, seems to always understand.
- (1) Seems to often understand.
- (2) Sometimes understands.
- (3) Rarely or never demonstrates understanding.

# Rate this child’s use of appropriate eye contact. Appropriate eye contact is eye contact that appears natural (i.e. not staring at a person the entire time, but making and disengaging eye contact naturally and flexibly). If the child appears shy, rate based on their behavior in the later part of the clip if they demonstrate improved eye contact. If eye contact never improves, rate accordingly.

- (0) Yes, always does this. Exhibits clear, flexible gaze that is linked with communication.
- (1) Often makes socially-directed eye contact.
- (2) Sometimes makes socially-directed eye contact.
- (3) Rarely or never makes socially-directed eye contact.

# Does the child look up and pay attention to the examiner or parent/caregiver when they talk to [him/her] without having to call the child’s name?

- (0) Yes, always pays attention without having to call name.
- (1) Often does this, but is sometimes distracted.
- (2) Sometimes does this, mixed with attention when name is called.
- (3) Rarely or never does this – child only pays attention if name is called (or doesn’t pay attention at all).

# Does the child display any developmental challenges? (A developmentally appropriate 3-year-old child can consistently use multiword phrases and carry on a conversation with 2-3 sentences. Follows 2-step directions, e.g. “pick up your coat and bring it to me.” Most speech is understandable. Sorts objects by shape and color. Imitates people or actions, and plays make-believe with toys or people.

**Climbs and runs well. A 2-year old uses 2-4 word sentences, follows simple instructions, begins sorting objects by color or shape, and copies adult’s actions.)**

- (0) Met all milestones. No behavior indicating developmental delay.
- (1) Uncertainty regarding behavior when compared with children the same age. Child may still be developmentally appropriate, but development is not clearly "normal” in the video to determine.
- (2) Some developmental delays are demonstrated, with some minor or moderate departures from established milestones.
- (3) Definite developmental delays, but not obvious as autism. Implies presence of another developmental delay.
- (4) Definite developmental delays that are clearly in line with autism symptoms.

# Based on what you’ve seen in this video, would the child comfort another person with words or actions if that person was not feeling well, hurt, or sad?

- (0) Yes, would definitely do this.
- (1) Probably would do this (50/50 chance).
- (2) Unlikely.
- (3) Almost certainly would not do this.

# Does the child seem to enjoy participating in social games and/or social interactions?

- (0) Yes, clearly enjoys being engaged in social activities.
- (1) Often does this, but limited.
- (2) Sometimes demonstrates enjoyment, but does not spontaneously participate in interaction.
- (3) Rarely or never demonstrates enjoyment in any social interaction.

# Does the child get upset, angry or irritated by particular sounds, tastes, smells, sights or textures?

- (0) Yes, always gets upset or irritated by sensory stimulation.
- (1) Often does this.
- (2) Sometimes does this.
- (3) Rarely or never does this.

# Does the child imitate others' actions, or would the child imitate others if given the chance?

- (0) Yes, would definitely do this.
- (1) Probably would do this (50/50 chance).
- (2) Unlikely that the child would do this.
- (3) Almost certainly would not do this.

# Can you tell from the look on [his/her] face that the child is happy, surprised, sad, angry, afraid, guilty or embarrassed?

- (0) Yes, always clearly expresses emotions in facial expressions.
- (1) Often expresses emotions.
- (2) Sometimes expressive.
- (3) Rarely or never demonstrates variation in facial expression.

# Does the child stare at objects for long periods of time or focus on particular sounds, smells or textures, or like to sniff things?

- (0) Yes, very drawn to sensory stimulation.
- (1) Often does this.
- (2) Sometimes does this.
- (3) Rarely or never does this.

# Does the child play pretend games when he/she plays with his/her toys?

- (0) Yes, always does this.
- (1) Often does this.
- (2) Sometimes does this.
- (3) Rarely or never does this.

# Does the child shake his/her head "no" or nod his/her head "yes" when asked a question or a request is made?

- (0) Yes, always does this.
- (1) Often does this.
- (2) Sometimes does this.
- (3) Rarely or never does this.

# Does the child like to share his/her excitement with others about things he/she likes or that make him/her happy? (for example: shows toy or drawing to parent)

- (0) Yes, always does this.
- (1) Often does this.
- (2) Sometimes does this.
- (3) Rarely or never does this.

# Rate the child’s responsive social smile.

- (0) Excellent: Clearly smiles in response to another person’s smile and/or playful verbal interaction.
- (1) Good: Smiles in response to another person’s smile/and or playful verbal interaction although stilted.
- (2) Satisfactory: Smiles only after physical interactions or upon request.
- (3) Poor: Does not smile at people but may smile at other things.

# Does the child show or deliberately orient an object to call other’s attention to things of interest

- (0) Yes. With accompanied eye contact.
- (1) Yes. Without accompanied eye contact.
- (2) No. The child has the opportunity to show or direct other’s attention but does not do so.

# Rate the child’s ability to draw another person’s attention to an object out of reach for the purpose of sharing information, and not requesting something (i.e. not that they want a snack, or to be picked up, etc.)

- (0) Excellent: Shifts gaze between object/person of interest to another person and back to the object/person (3- Point Gaze)
- (1) Satisfactory: Shifts gaze between object/person of interest to another person but not back to object/person (2- Point Gaze)
- (2) Poor: Does not show spontaneous joint attention.

# Rate the child's play with objects (includes utensils, crayons, etc.).

*Clarification: Is the child playing appropriately or inappropriately with toys and/or objects?*

- (0) Excellent: Clear spontaneous appropriate play.
- (1) Good: Some spontaneous appropriate play.
- (2) Satisfactory: Some appropriate play but does not engage spontaneously.
- (3) Poor: Stereotyped or inappropriate play only.

# Rate the child’s creativity in his or her play and/or conversations

- (0) Excellent: Clear demonstration of creativity.
- (1) Good: Some demonstration of creativity
- (2) Satisfactory: Some demonstration of creativity but not spontaneous.
- (3) Poor: Does not demonstrate creativity.

# Does the child use stereotyped words or phrases? Stereotyped words could include repeating lines from a movie, or repetitive language that is inappropriate in the social context. Odd uses of words or

**phrases include using the incorrect pronoun (“she/he” instead of “you”), made-up words, sentences that don’t make sense in the context, or referring to oneself in the third person.**

- (0) Rarely or never uses stereotyped language.
- (1) Sometimes uses stereotyped language.
- (2) Often uses odd words or stereotyped language, but not all language is stereotyped.
- (3) Almost always uses odd or stereotyped language.

# Rate the child's spontaneous gestures

*Clarification: Descriptive gestures (e.g., acting out rinsing a toothbrush or showing how a roller coaster curves through the air), Conventional gestures (e.g. thumbs up, waving hello), Instrumental gestures (e.g., shrugging, head nodding, head shaking). Exclude pointing and emphatic gestures (e.g., foot stomping). Descriptive gestures = acting out an action, doesn’t have to be meshed with language. Conventional gestures = waving hello/goodbye, Informational gestures = how old are you, holds up 3 fingers/how big is that dog, moves arms to show how big Instrumental gestures = raising a hand, raising arms to get picked up Include: Spontaneous gestures, unprompted but can be in response to a question. Exclude: Pointing*

- (0) Excellent: Spontaneous use of at least one descriptive gesture. Conventional and instrumental gestures may be present as well.
- (1) Good: Spontaneous use of more than one conventional and/or instrumental gesture but no descriptive gestures.
- (2) Satisfactory: Spontaneous use of one conventional or instrumental gesture but no descriptive gestures.
- (3) Poor: Does not spontaneously use gestures.

# Rate the child’s ability to indicate enjoyment with others

- (0) Excellent: Clearly expresses contentment in interactions with others.
- (1) Satisfactory: Expresses enjoyment in interactions with others although limited in amount or quality.
- (2) Poor: Does not express enjoyment in interaction with others but may express enjoyment/satisfaction in his/her own actions or does not show interest in others around them.

# Rate the quality of the child's initiations of social interactions. Can be verbal or nonverbal (i.e. raising arms to ask for hug).

- (0) Excellent: Initiation of social interaction appropriate to context.
- (1) Good: Sometimes odd initiation of social interactions.
- (2) Satisfactory: Initiations are often inappropriate to the social context.
- (3) Poor: Child does not initiate any social interactions.

# Does the child display hand and finger and other complex mannerisms, such as hand tensing, hand flapping, or holding up hands and looking intensely through the finger gaps?

- (0) No mannerisms are observed.
- (1) Yes, brief or unclear mannerisms.
- (2) Yes, clear mannerisms observed.

# Does the child display inflexible stereotyped interests (focuses only on subject of interest to them, spinning wheel on toy, lining up toys) or repetitive behaviors (i.e. rocking, spinning, tapping)?

- (0) No. Repetitive or stereotyped behaviors not observed.
- (1) Yes. Behaviors observed briefly.
- (2) Yes. Behaviors clearly observed but attention is also directed to other objects or activities.
- (3) Yes. Behaviors observed the entire time. The child is rigid in his/her focus on the interest or behavior and may become upset if interrupted.

# Do you think the child has autism?

- (0) No, I am confident the child does not have autism
- (1) No, but I am unsure
- (2) Yes, but I am unsure
- (3) Yes, I am confident the child has autism

**File S1**. Full list of behavioral features captured.

# 1. Rate this child’s display of echolalia (does the child immediately repeat the last statement made by the parent/caregiver, for example, repeating the parent’s question? Do not include repetition that is appropriate to use in conversation or that is requested by the parent, i.e. “Say, ‘dog.’”).

- (0) No demonstration of echolalia. Does not repeat others’ speech.
- (1) Occasionally or rarely echoes others.
- (2) Mixed: some regular echoing of words and phrases, but also some language.
- (3) Mostly echoed speech

# 2. Rate this child’s expressive language/ conversation ability:

*Clarification: Expressive language is words used to communicate to others. Consider appropriate verbal skills for age when rating this question.*

- (0) Excellent: Child is able to have a back and forth conversation, child responds to questions, and elaborates on their responses. Child may spontaneously address another person to engage in conversation.
- (1) Good: Child is able to respond to questions from examiner AND elaborates on their responses sometimes. Child engages individual(s) in conversation at times. Child elaborates and engages with individual(s) during conversation, but somewhat less than would be expected for their expressive language level and/or age.
- (2) Satisfactory: The child maintains very little back and forth conversation. Child may only talk about things they are interested in, and not respond and stay on topic with others speaking to them. Child may address others spontaneously, but there is little engagement in back and forth conversation from the child.
- (3) Poor: The child rarely or never attempts to communicate with others in the video. Child may speak often in the video, but most of their language is not aimed at communicating with others (i.e. may be echoed speech, or the child may speak to themselves). Child may make some limited responses to the examiner (for example, saying “yes” or “no” with no elaboration).

# Rate this child’s use of appropriate eye contact. Appropriate eye contact is eye contact that appears natural (i.e. not staring at a person the entire time, but making and disengaging eye contact naturally and flexibly). If the child appears shy, rate based on their behavior in the later part of the clip if they demonstrate improved eye contact. If eye contact never improves, rate accordingly.

- (0) Yes, always does this. Exhibits clear, flexible gaze that is linked with communication.
- (1) Often makes socially-directed eye contact.
- (2) Sometimes makes socially-directed eye contact.
- (3) Rarely or never makes socially-directed eye contact.

# Can you tell from the look on [his/her] face that the child is happy, surprised, sad, angry, afraid, guilty or embarrassed?

- (0) Yes, always clearly expresses emotions in facial expressions.
- (1) Often expresses emotions.
- (2) Sometimes expressive.
- (3) Rarely or never demonstrates variation in facial expression.

**Child age.**

**File S2**. Questions and features used in LR5 classifier. The LR5 uses the human annotations for four features and the child’s age as the fifth feature.

# 2. Rate this child’s expressive language/ conversation ability:

*Clarification: Expressive language is words used to communicate to others. Consider appropriate verbal skills for age when rating this question.*

- (0) Excellent: Child is able to have a back and forth conversation, child responds to questions, and elaborates on their responses. Child may spontaneously address another person to engage in conversation.
- (1) Good: Child is able to respond to questions from examiner AND elaborates on their responses sometimes. Child engages individual(s) in conversation at times. Child elaborates and engages with individual(s) during conversation, but somewhat less than would be expected for their expressive language level and/or age.
- (2) Satisfactory: The child maintains very little back and forth conversation. Child may only talk about things they are interested in, and not respond and stay on topic with others speaking to them. Child may address others spontaneously, but there is little engagement in back and forth conversation from the child.
- (3) Poor: The child rarely or never attempts to communicate with others in the video. Child may speak often in the video, but most of their language is not aimed at communicating with others (i.e. may be echoed speech, or the child may speak to themselves). Child may make some limited responses to the examiner (for example, saying “yes” or “no” with no elaboration).

# Rate this child’s speech patterns.

*Clarification: Abnormal speech includes: a*bnormal volume, pitch, intonation, rate, rhythm, stress, prosody or volume in speech. Consider appropriate language abilities for the child's age while rating.

- (0) Child has no abnormal qualities to speech and has appropriate volume, intonation, rhythm, rate, pitch, etc. when speaking.

o (1) Child has slightly unusual qualities to speech. For example, speaking too softly or too loudly, monotone or exaggerated intonation at times, speech that is too fast or too slow.

- (2) Child’s speech is abnormal. The child’s speech patterns make them hard to understand, due to odd volume, tone, rate/rhythm, intonation. Speech could be extremely monotone, pitch that seems unnatural (too high or too low), rhythm and pitch that seems robotic, or overly animated.
- (3) Stutter or stammer, and probable speech delay/disorder.

# Rate this child’s change in gaze, facial expression, and gesture while the child vocalizes. Include vocalizations used to maintain interaction or to respond to a person, as well as initiations

- (0) Child’s vocalization is paired with appropriate facial expression, gesture, gaze.
- (1) Child’s vocalization is paired with abnormal, limited frequency and/or range of gesture, gaze and facial expression. Child may only rely on only gesture, or only gaze, or only vocalization.
- (2) Child rarely or never links their vocalization with nonverbal communication, i.e. no or minimal use of gestures, facial expression, or socially directed gaze.
- (3) Child may avoid direct gaze, but could be attributed to shyness. Shows some use of nonverbal communication linked with vocalization.

# Rate the child’s self-injurious or aggressive behaviors towards themselves. This includes behaviors such as biting his/herself, hitting his/her head, or pulling his/her hair.

- (0) Does not harm him/herself.
- (1) Unclear. Some possible self-injury, and/or very rare self-injury.
- (2) At least one clear example of self-injury.

# Can you tell from the look on [his/her] face that the child is happy, surprised, sad, angry, afraid, guilty or embarrassed?

- (0) Yes, always clearly expresses emotions in facial expressions.
- (1) Often expresses emotions.
- (2) Sometimes expressive.
- (3) Rarely or never demonstrates variation in facial expression.

# Does the child like to share his/her excitement with others about things he/she likes or that make him/her happy? (for example: shows toy or drawing to parent)

- (0) Yes, always does this.
- (1) Often does this.
- (2) Sometimes does this.
- (3) Rarely or never does this.

# Does the child use stereotyped words or phrases? Stereotyped words could include repeating lines from a movie, or repetitive language that is inappropriate in the social context. Odd uses of words or phrases include using the incorrect pronoun (“she/he” instead of “you”), made-up words, sentences that don’t make sense in the context, or referring to oneself in the third person.

- (0) Rarely or never uses stereotyped language.
- (1) Sometimes uses stereotyped language.
- (2) Often uses odd words or stereotyped language, but not all language is stereotyped.
- (3) Almost always uses odd or stereotyped language.

# Rate the quality of the child's initiations of social interactions. Can be verbal or nonverbal (i.e. raising arms to ask for hug).

- (0) Excellent: Initiation of social interaction appropriate to context.
- (1) Good: Sometimes odd initiation of social interactions.
- (2) Satisfactory: Initiations are often inappropriate to the social context.
- (3) Poor: Child does not initiate any social interactions.

# Does the child display inflexible stereotyped interests (focuses only on subject of interest to them, spinning wheel on toy, lining up toys) or repetitive behaviors (i.e. rocking, spinning, tapping)?

- (0) No. Repetitive or stereotyped behaviors not observed.
- (1) Yes. Behaviors observed briefly.
- (2) Yes. Behaviors clearly observed but attention is also directed to other objects or activities.
- (3) Yes. Behaviors observed the entire time. The child is rigid in his/her focus on the interest or behavior and may become upset if interrupted.

**Child age.**

**File S3**. Questions used in LR10 classifier. The LR10 uses the human annotations for nine features and the child’s age as the tenth feature.

The first HIT in the filtering process asked workers to guess the clinical diagnosis of children based on 10 short videos. The possible diagnoses for each video were Obsessive Compulsive Disorder (“OCD”), “Apraxia”, Attention Deficit Hyperactivity Disorder (“ADHD”), “Speech Delay”, and “No Condition”. Workers were also asked to provide a short free-form response for their answer. We progressed workers to the next round of HITs if (1) the free-form response to the selected answer was reasonable as determined by the authors, (2) the workers spent at least 20 seconds per video on average, (3) the workers answered correctly on at least 5 of the 10 videos. Workers who passed this round of filtering were invited to the next round of HITs through a small worker bonus of $0.05 and a corresponding message detailing the next steps for the task. These workers were provided a custom MTurk qualification enabling them to see the next set of HITs.

In the next round of filtering HITs, a task identical to the one used in the primary study was presented but with a set of 5 videos not included in the primary study. In this task, an unstructured video of a child with a potential developmental delay interacting with his or her parent was displayed. Following was a series of 31 multiple choice questions with wording corresponding to features extracted in prior work via ML analysis of electronic health records generated by the clinical use or clinical administration of those tools. Due to the short video length, several of the behaviors asked in the questionnaires were not exhibited in any particular video. We hypothesized that select crowd workers would be capable of accurately and precisely inferring a categorical estimate of the child’s behavior in other situations based solely on the video clip, resulting in a “*human imputation*” method for missing behaviors. We therefore did not provide any “N/A” option for any multiple question, regardless of whether information about the behavior in question was present within the video. Workers were filtered out based on the mean deviation from the answers provided by researchers on the categorical answers. In addition, workers were filtered by quality control metrics such as the number of blank answers and the time spent working on the task. Workers progressing to the next round were similarly provided with a custom MTurk qualification, bonus, and message.

In the final round of filtering HITs, more tasks identical to the one used in the primary study were given to workers. Workers were filtered by the quality control measures above as well as their deviation from other workers answering the same question for the same video. Workers who provided blank answers had their HITs rejected but were given an opportunity to revise their answer. If they revised, they were not filtered out and their HIT was approved. If no response was provided, the worker was filtered out. Workers were evaluated based on their classification performance against the gold standard classifier described above. Workers whose features consistently led to a prediction differing from the gold standard classifier were filtered out. As in the previous rounds, workers selected to participate in the final study were provided with a custom MTurk qualification and recruitment message.

**Method S1.** Detailed description of crowd selection process.

To understand the effect on parental trust of privacy-preserving mechanisms on videos of children with autism, we recruited 510 parents on MTurk to conduct a survey about willingness to share videos of their children under various privacy conditions. We showed parents a video of a child and asked them whether they would share the video with other workers on MTurk under various privacy conditions (see *Materials and Methods: Survey of parents* for details). We find that out of the 510 parents surveyed, 144 are not fine with sharing unaltered videos of their child but are fine with sharing videos with the face box and pitch shift. 137 parents are not fine with sharing the unaltered video but are fine with sharing the box. Only 12 parents were not fine with unaltered videos but were fine with only pitch shifted videos. Interestingly, 125 parents were fine with sharing unaltered videos but no longer expressed a desire to share videos with pitch shift and face obfuscation applied. To understand the reasoning for the aversion to sharing privacy-preserved videos in comparison to unaltered videos, we categorized the free-form text explanations provided by parents for each of their responses. 47 parents were not willing to share when visual and audio privacy mechanisms were applied because privacy concerns remained when sharing the video; 31 parents expressed that the content in the video was inappropriate for public sharing; 63 parents expressed that the privacy mechanism removes the useful qualities of the video for research purposes; 85 stated that the privacy makes the video no longer desirable for public sharing; 8 stated that the video would no longer be useful for research purposes (see *Supplementary Information: Table S1*). These results suggest that a substantial portion of parents are more likely to share videos of their children with crowd workers when privacy-preserving alterations are made, but other parents would no longer be content with sharing the altered videos. In total, 420 of the 510 parents surveyed were willing to share results for at least one of the video conditions presented.

All privacy survey participants were vetted by the MTurk system to be parents using Amazon’s filtering system. The survey displayed a single video of a child under each of the four privacy conditions (unaltered, box covering the face, pitch shifted down by two semitones, and both a box covering the face and the pitch shifted down). For each video, we asked, “*Would you be willing to share a similar video of your child with other workers on MTurk if you knew the purpose was to help pediatric research for developmental delays?*”. Workers could select either “Yes” or “No”. We then provided a required free form text box where we asked workers, “*Provide your reasoning below*”. The quality of these free form responses was used to filter out responses. If the answer had nothing to do with the questions asked, we rejected the response from the worker. We aimed to continue collecting survey data until 500 survey responses were collected. Due to quality control filters applied to survey responses, 510 valid survey responses were collected in total.

**Method S2.** Method and results for crowdsourced survey of parents about sharing video with and without privacy alterations.
